# An Encoder–Decoder Architecture within a Classical Signal-Processing Framework for Real-Time Barcode Segmentation

**DOI:** 10.3390/s23136109

**Published:** 2023-07-03

**Authors:** Óscar Gómez-Cárdenes, José Gil Marichal-Hernández, Jung-Young Son, Rafael Pérez Jiménez, José Manuel Rodríguez-Ramos

**Affiliations:** 1 Department of Industrial Engineering, Universidad de La Laguna, 38200 La Laguna, Spain; ogomezca@ull.edu.es (Ó.G.-C.); jmramos@ull.edu.es (J.M.R.-R.); 2 Biomedical Engineering Department, Konyang University, Nonsan-si 320-711, Republic of Korea; jyson@konyang.ac.kr; 3 Institute for Technological Development and Innovation in Communications, Universidad de Las Palmas de Gran Canaria, 35017 Las Palmas, Spain; rafael.perez@ulpgc.es; 4 Research & Development Department, Wooptix S.L., 38204 La Laguna, Spain

**Keywords:** Radon transform, scale-space methods, multiscale DRT, barcodes, encoder–decoder, pixelwise segmentation, classical signal processing

## Abstract

In this work, two methods are proposed for solving the problem of one-dimensional barcode segmentation in images, with an emphasis on augmented reality (AR) applications. These methods take the partial discrete Radon transform as a building block. The first proposed method uses overlapping tiles for obtaining good angle precision while maintaining good spatial precision. The second one uses an encoder–decoder structure inspired by state-of-the-art convolutional neural networks for segmentation while maintaining a classical processing framework, thus not requiring training. It is shown that the second method’s processing time is lower than the video acquisition time with a 1024 × 1024 input on a CPU, which had not been previously achieved. The accuracy it obtained on datasets widely used by the scientific community was almost on par with that obtained using the most-recent state-of-the-art methods using deep learning. Beyond the challenges of those datasets, the method proposed is particularly well suited to image sequences taken with short exposure and exhibiting motion blur and lens blur, which are expected in a real-world AR scenario. Two implementations of the proposed methods are made available to the scientific community: one for easy prototyping and one optimised for parallel implementation, which can be run on desktop and mobile phone CPUs.

## 1. Introduction

One-dimensional barcodes are a means for visually encoding information to make it machine readable, making use of alternating bars and spaces of different widths [1].

This paper deals with barcode segmentation for camera-based barcode readers. To reduce user assistance in aligning the reader with the codes, segmentation produces an image mask that indicates exactly in which pixels, if any, barcodes are present, thereby indicating where the next stage, that is decoding the data encoded in the barcode, should take place.

The localisation and proper segmentation of barcodes within an image have been frequently addressed in the literature, but not so frequently when augmented reality (AR) [2] goals are also set. For the real-time augmentation of a device’s camera stream, the entire barcode localisation/segmentation and decoding pipeline must fit within a few milliseconds. If the identification of the code in a frame on sight is transferred to the user with any latency, the augmented experience becomes unfeasible due to the misalignment of the marks in the augmented world and their real-world equivalents. In this sense, even if a method achieves high detection accuracy, it is not suited to AR applications if it does not keep the latency low.

### 1.1. Barcode Detection Summary

Up until a decade ago, most of the proposed methods could be classified as classical [3,4,5,6,7,8,9,10,11] or based on machine learning, i.e., the latest trend.

In classical methods, the main underlying idea is that the gradient in a zone of parallel bars shares a common preferential orientation. Thus, in the absence of rotation, when the camera and code are aligned, areas with high horizontal gradients and low vertical gradients can be classified as one-dimensional barcoded areas. This main idea can be complemented using methods to achieve rotation invariance by detecting the orientation of the codes, for example by analysing image patches with Radon or Hough transforms; contrast invariance can be achieved using adaptive or histogram thresholding; further, in order to delimit the code region, a mixture of thresholding, connected components, and/or morphological operations are used to finish off the contours of the codes.

Other classical methods rely on a geometrical line or bar detector [12,13,14,15]. In the case of two-dimensional codes, which are not the subject of this work, the gradient criterion is not sufficient, so edge detection is replaced by corner detection [9].

Since 2013 [15,16,17], these classical techniques have been hybridised with neural networks, either to replace computationally expensive line transforms for angle detection or to better contour the barcode shape.

In recent years, there has been a renewed interest in the subject, with several papers applying advances in machine learning to the problem at hand, borrowing generic networks and retraining them to solve the specific problem of barcode localisation [18,19]. In this latter group of articles, the results are quite good—comparable to those of the best classical approaches—both in terms of detection accuracy and inference time, but also quite similar among the different proposed methods [20].

### 1.2. Semantic Segmentation

Recently, there has been an enormous amount of contributions in the field of object recognition [21], and a new type of **c**onvolutional **n**eural **n**etwork (CNN) has emerged to address the problem of semantic segmentation at the pixel level. The problem that these networks solve consists of assigning a label to each pixel in the image, indicating that it is part of an object of a certain class. CNNs have become better and better at solving this problem lately [22].

**Figure 1:**
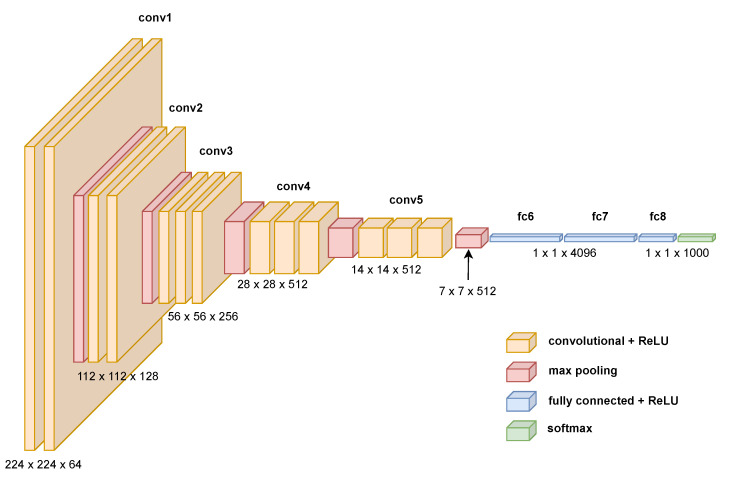
Topology of the VGG16 model. ReLU stands for the type of activation function used: rectified linear unit.

**Figure 2:**
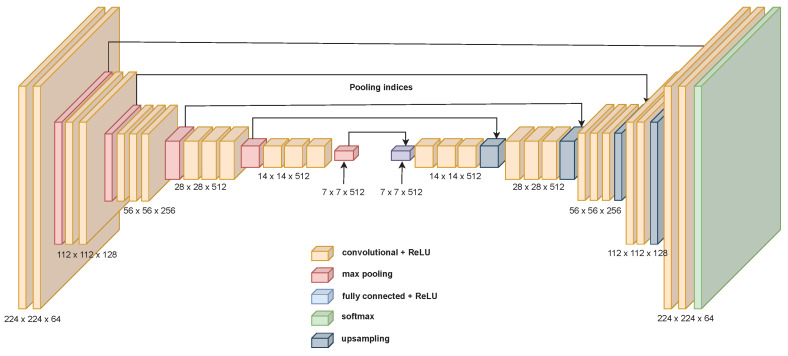
SegNet topology. It uses VGG16 as the encoder and adds a specularly symmetric decoder. Note that the indices of the max pooling operations are used when upsampling.

These networks usually have some kind of symmetry. The first part of the network, the encoder, starts with the input size of the image and lowers its spatial dimensions while increasing the depth of the data size, as shown, for example, in Figure 1. The second part of the network performs a reversed path, where the spatial dimension increases and the depth decreases again. A good example of this is the SegNet network [23], which utilises the VGG16 network as the encoder and adds a new decoder that enables the network to perform pixelwise semantic segmentation, as shown in Figure 2.

The result of this kind of topology is that not only the image is classified into classes, effectively recognising the objects that are present as VGG16 does, but also the object that lies behind each pixel is labelled with great accuracy, and the labels are correctly constrained to the borders of the object.

### 1.3. Review of Selected Works

In this subsection, we provide a more detailed analysis of a selection of previous contributions.

Robust angle-invariant, 1D barcode detection [16]

One of the most-relevant papers in barcode recognition—dating from 2013—is Zamberletti et al.’s, who pioneered the use of machine learning and line domain transforms in angle detection, targeted mobile phones as their platform for combined capture and computation, and established one of the publicly available datasets still in use to assess detection accuracy. Their technique consists of a concatenation of tasks: Canny detection for edge enhancement, Hough transform to detect lines, a multilayer perceptron operating on the angular dimension of the Hough domain to detect the presence of barcodes, and histogram-guided selection in the displacement dimension to determine bounding boxes. Despite its low throughput—each image takes 270 ms to be processed on a mobile phone—its rotation invariance achieved by using the Hough transform is unprecedented. Thanks to the use of machine learning, the technique is also capable of detecting partially occluded barcodes, setting the state of the art at the time, correctly detecting around 83% of barcodes in labelled datasets.

Real-time barcode detection in the wild [10]

Creusot and Munawar, in 2015, used maximal stable extremal region to detect, filter, and cluster bars pertaining to barcodes. They also used the Hough transform in the clustering stage. They increased the accuracy obtained by Zamberletti et al. [16] by 10 percent and, by moving to a desktop PC, could process a frame of 640 × 480 in the order of 100 ms.

Low-computation egocentric barcode detector for the blind [11]

The same authors, a year later, aiming to process frames obtained with a wide-angle video camera, discarded their previous method because it was not resistant to motion blur and came up with a new method consisting of a geometric algorithm to detect parallel lines, where a representative line of the cluster determines the height of the barcode bounding box and an estimator of variations in the bisector of the line accounts for the width of the box. This new method increased by up to 98% the capacity to detect codes on simple datasets and lowered the time cost of processing a frame to 40 ms. However, on a more realistic dataset of images taken with a high-resolution video camera—which they created and called EGO, but which is not publicly available—the detection rate dropped to 60%, and the computation time on a PC increased to 116 ms. However, this could be considered the best approach to barcode segmentation with classical methods.

Real-time barcode detection and classification using deep learning [18]

In 2017, Hansen et al. used a recently developed deep learning object detection algorithm, You Only Look Once, with the Darknet19 network structure on 416 × 416 images. As the YOLO output consists of rectangles containing barcodes, which can appear rotated, they used another network to predict the rotation angle. They obtained the same accuracy as Creusot and Munawar [11], but thanks to the use of a GTX 1080 GPU, they could process a frame in 14 ms. However, this method cannot be considered to have processing time comparable to video acquisition time on CPUs.

Universal barcode detector using semantic segmentation [17]

More recently, Zharkov and Zagaynov, following the advances in semantic segmentation, specifically designed a neural network algorithm and trained it to detect 16 different types of one-dimensional barcodes and 5 types of two-dimensional barcodes. Its accuracy was on par with that of the method by Creusot and Munawar [11] on one-dimensional barcodes. Its network structure, much more trimmed than YOLO’s, achieved the processing time of only 3.8 ms on a GPU; however, when employed on a desktop CPU, computation took 44 ms on 512 × 512 images, which is the same time taken by the best of the classical methods. Though it is close, it is not in real time, even on low-resolution images.

One-dimensional barcode detection: novel benchmark datasets and comprehensive comparison of deep convolutional neural network approaches [24]

Following another work by the same group [20], where they systematically analysed the literature on neural networks applied to the problem under discussion, the authors created two training datasets: one containing consumer goods codes and the other containing postal labels. Both were created based on real, uncontrolled environments. They then trained and tested a well-known algorithm, Faster R-CNN [25], as well as four underexplored ones, EfficientDet [26], RetinaNet [27], YOLO v5 [28], and YOLO x [29]. They concluded that the YOLO v5 algorithm performed better in terms of accuracy and runtime on most datasets. Unfortunately, the authors did not provide the inference time on CPUs.

### 1.4. Remaining Challenges

Despite such a proliferation of articles, several challenges remain unaddressed.

Success in training neural networks heavily depends on the quality of the set of images they are trained on, and these have usually been taken as sharp, still pictures under good lighting conditions. In some cases, they have even been synthetically generated by overlapping undistorted barcodes on real images [30,31].

Publicly available labelled barcode datasets usually have small dimensions, e.g., 640×480 pixels [16], and since the computational requirements markedly increase with image size in the training phase, those sizes have been accepted as good enough. In addition, this is justified by the fact that barcodes so small that they cannot not be detected at that scale would also not be decodable. Nevertheless, in AR, barcode detection is desirable even at non-decodable scales.

More importantly, it is neglected that a barcode reader designed to work in AR applications is not limited to analysing static and well-exposed images but also analyses short-exposure image sequences. Moreover, when the camera approaches the code, until the movement stabilises, most frames exhibit motion blur. In addition, lens blur is frequent when a user starts to aim at a barcode and the lens has not had time to lock focus or when there are objects at different focus distances. However, out-of-focus images have generally not been of interest in previous works, which is again collaterally justified by the impossibility of decoding them.

Another problem is that quality assessment often relies on metrics that, despite being objective and repeatable, do not necessarily explain the goodness of the method in real-world scenarios. The often used Jaccard index, or the similar Dice coefficient, measures the overlapping percentage between the ground truth barcode region and the predicted bounding boxes. Probably induced by machine learning competitions [32], it is assumed that a higher Jaccard index means better performance. However, in a real-use case, a raster line good enough to perform decoding can be extracted from a predicted bounding box with a low Jaccard index. To our knowledge, no study has been conducted to determine the Jaccard index threshold needed to retrieve a satisfactory bisector line. Instead, most neural network papers provide detection metrics for various Jaccard index thresholds; virtually all achieve perfect detection rates at lower index values. The more barcodes can be decoded at those low thresholds, the lower the benefits of improving accuracy at high thresholds are. However, it is at these thresholds, close to perfect overlapping, that diminishing improvements are being reported in the state of the art.

On the other hand, very few works claim to achieve computation time lower than 33 ms, as required by AR applications. Moreover, when they do, it is by running them on powerful desktop GPUs, which are not available in a real AR deployment.

The last issue is that the evaluation of the methods lacks repeatability in the absence of the actual implementation code. This leads to the fact that some of the methods that are considered slow today could probably favourably compare to the latest methods if run on modern platforms. Because of this, it is not possible to repeat quality assessment with metrics other than those proposed by the authors.

### 1.5. Purpose of This Work

To overcome the challenges mentioned above, this paper revises a modification of the discrete Radon transform [33], which makes the local detection of bar-shaped structures at a certain scale possible. This method presents a trade-off between the density of the grid on which it returns the results and the accuracy with which it determines the existence and orientation of the bars. This method works locally on non-overlapping image tiles; therefore, the spacing, or stride, between tiles is equal to the tile size.

In this contribution, the above method was improved to achieve multiscale detection. Multiscale methods [34] can help by serving two purposes: barcode patterns are identified with scale invariance—which is important in a scenario where the operator can freely zoom the reader in and out of the codes—and computation time is reduced by working at each scale with the appropriate scale size, which is usually a fraction of the input resolution.

The following tasks were tackled, with an emphasis on reducing computation speed, and thus latency, to make AR feasible without degrading the quality of segmentation:

1. To propose an adapted version of the above method to allow overlapping among local detection zones so that tile sizes and spacing between detection processes are decoupled.
2. To propose a second adapted version that works by merging the multiple scales of the original method, i.e., the different tile sizes, in an automatic and optimal way.
3. To perform a comparison between the original method and the two cited innovations, and one between these and existing methods for barcode segmentation.

The following section presents the ideas behind the existing method that are necessary to realise the proposed methods. Then, the outline of the paper is given at the end of Section 2.3.

## 2. Discrete Radon Transform as a Bar Detector

### 2.1. Discrete Radon Transform

The Radon transform [35] makes it possible to study a two-dimensional function f(x,y) in terms of the integrals across all the lines that pass through it. The parameters that define the different lines are slope and displacement, or the interception of the line with the axis.

In the 1990s, several authors [36,37,38] simultaneously proposed a discrete Radon transform (DRT), which exhibits linearithmic complexity, $O(N^{2}\log N)$, by expressing the line integrals as summations over a stripe of connected samples of unit width and approximately constant slope over discrete signals. The virtue of the method is to work with “loose digital lines” that do not require interpolation. Moreover, these lines are constructed recursively with a multiscale approach, where the sums of segments of length two are carried out only once; when segments of size four are constructed, it is performed by optimally reusing the partial results of the previous scale, and so on. The result, for an input of size N×N, is obtained after $\log_{2}N$ stages. In this method, the partial results are a means to arrive at the summation over the full domain and are discarded, but now, they will be essential to our purposes.

Another remarkable characteristic of the DRT is that the lines are expressed in the form y=x·slope+displacement, with the slope value being between 0 and 1, so the basic algorithm can only solve a quadrant consisting of $45^{\circ }$. If the input is vertically inverted, the result then corresponds to y=−(x·slope+displacement). Similarly, when adding a transposition, the eminently vertical lines are obtained. Thus, after running the basic quadrant algorithm on three additional transpositions and/or the flipping of the input, the $180^{\circ }$ needed to cover the angular dimension of the slope is obtained.

Two modifications to the original DRT algorithm are particularly relevant to the subsequent discussion and elucidation of the new methods:

1. A method to better expose the parallelism of the DRT [39], which achieves, in one pass, the eminently horizontal lines of the form y=±(x·slope+displacement), and in another pass, those of the form x=±(y·slope+displacement), thus changing from four quadrants to two groups of 90 degrees (around the horizontal axis and the vertical axis). Additionally, this reformulation eliminates the need for prior zero padding, which the conventional DRT requires.
2. A bar detector method, built upon the aforementioned modified DRT, that acts locally [33], giving an estimate of the presence of bars and their inclination for each block of size $2^{t}\times 2^{t}$ into which an image can be subdivided without overlapping. This local size is denoted by tile_size.

The second method is described in a more detailed manner in the following subsection.

### 2.2. From a Line Detector to a Local Bar Detector

**Figure 3:**
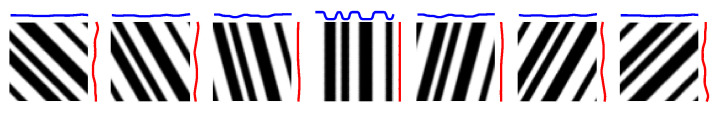
A series of rotated image tiles containing bars, surrounded by plots of their pixel intensity sums per row (red) and per column (blue). The variance difference among them is maximal when the bars are fully vertically or horizontally oriented.

An image region containing bar structures is characterised by the fact that the intensity of the pixel traversal at the angle at which the bars point is constant as we move longitudinally, either on a bar or in a space between bars. On the other hand, the intensity variation is maximal in the orthogonal direction, as we alternately traverse bars and spaces as fast as possible. This property seems to indicate that a bar detector must always consist of a gradient estimator, but this characteristic of bar structures can be expressed in another, equivalent way: If what is observed are the line integrals in the direction coincident with the bars, there is large variance between neighbouring line integrals (the result of accumulating the intensity of the bars at some displacements and the intensity of the spaces at nearby displacements), whereas in the orthogonal direction, all displacements cross both bars and spaces to the same extent; therefore, the variance between neighbours is low. See Figure 3.

**Figure 4:**
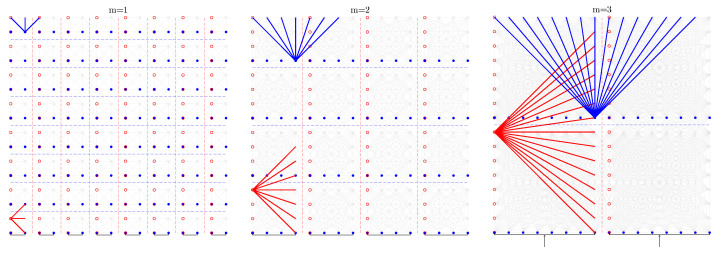
Illustration of how adjacent pixels and subsequently line segments are combined in the partial stages of DRT. In this example, 16×16 pixels (represented by circles) are combined initially, to the left, two by two; in the next stage, four by four; and finally, eight by eight. The final tile spacing between solutions (arrows, at the bottom) is eight. In each partial-stage solution, pixels are combined to consider each displacement and slope, but in non-overlapping vertical and horizontal bands, separated by dashed lines. Eminently horizontal lines are rendered in red, whilst vertical ones are rendered in blue.

The method described by Oliva-García et al. [33], consisting of the above method, works because the partial *m* stage of the modified DRT as described by Gómez-Cárdenes et al. [39] computes the sums at $2^{m+1}-1$ possible angles of line segments of length $2^{m}$, starting at each position $\left(2^{m}\cdot k_{x},d_{y}\right)$ with $d_{y}\in \left\{0..N_{y}-1\right\}$ but $k_{x}\in \left\{0..\frac{N_{x}}{2^{m}}\right\}$ for eminently horizontal lines and similarly for vertical ones. Note that in each partial stage *m*, since no overlapping is allowed, the solutions are $2^{m}$ apart from each other, as shown in Figure 4. The goodness of the method lies in the fact that the images themselves are not sheared to different angles and then columnwise and rowwise summed, but the very internal structure of the partial DRT stages, normally discarded as an intermediate product, consists precisely of the pixels accumulated by angle and displacement; this means that the values plotted in blue and red in Figure 3 can be found, just in another order, in the same colours in Figure 4.

Mixing the above two illustrative images, Figure 4 can be understood as the first three partial stages of the DRT on a problem of size N=16. If the process is stopped in this stage, the line integrals can be compared around the centres of the four tiles of size 8×8, which the DRT inner stages have created. The method returns the angle argument of the maximum variance differences between the horizontal and vertical lines within each tile, which are represented in red and blue, respectively, in the figure, supplemented with an activation metric that combines both variances.

The estimations at each scale are non-overlapping; the evaluations can only be given for sets of *T* neighbours separated by *T* from each other in each dimension. In turn, the number of evaluable angles also depends on the tile_size, since in areas of width *T*, there are *T* positive and *T* negative slopes that can be evaluated, giving a total of 2T. If exclusively considering horizontal lines, the number becomes 2T−1 when the double occurrence of the common ±0 slope is subtracted. Since they are to be compared with their vertical opposites, one more combination has to be subtracted due to coincidence and then the number has to be doubled (since the same comparison determines the existence in one direction and its orthogonal), up to a total of 2(2T−2)=4(T−1).

Importantly, with this method, if the scale of analysis—the tile_size—matches the scale of the bars and as long as the whole tile consists of an area containing a barcode, the method executes detection and returns the angle that is most coincident with the direction of the bars.

**Figure 5:**
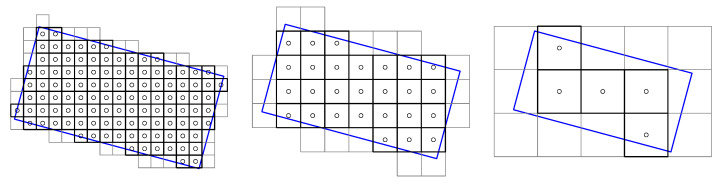
Dilemma of how to choose the right tile size to study a barcode region (in blue). From left to right, the tile subdivisions are shown for three different tile sizes, each of which quadruples the previous one in area. The tiles are depicted as rectangles with a circle at their centre and define the spacing of the output grid. The tiles whose centres are within the barcode region are depicted in a coarser line.

However, similar to other pattern-recognition problems, the scale is unknown a priori, and if it is not appropriately chosen, detection does not occur. Figure 5 symbolises the relationships that can take place between a barcoded area, represented by the blue rotated rectangle, and three possible scales. At smaller scales, as already emphasised, fewer angles are evaluated, but the grid of results is denser, and vice versa. Furthermore, the larger the scale is, the less guarantee there is that the tiles show areas that belong entirely to a barcode.

#### 2.2.1. Disadvantages of Working at a Single Scale

**Figure 6:**
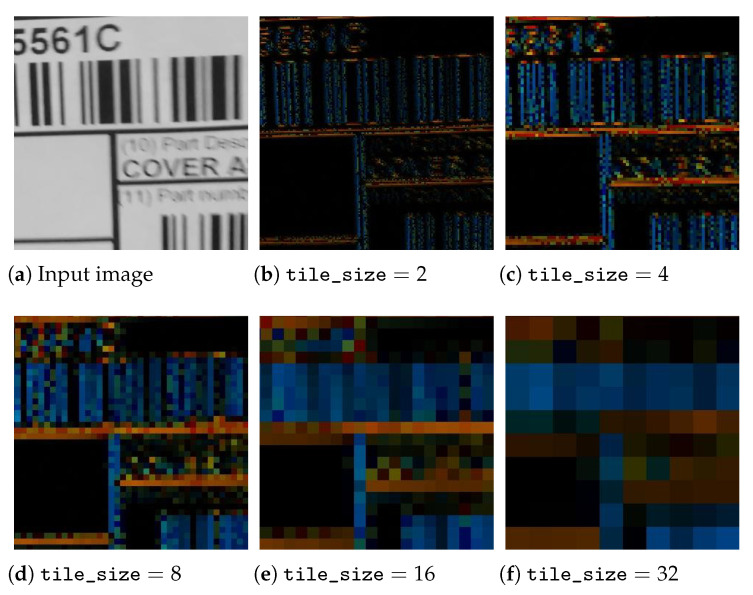
From left to right, from top to bottom: the output of the method by Oliva-García et al. [33] given the zoomed example image in the top-left corner at scales ranging from tile_size = 2 to tile_size = 32. Note that as the tile_size is doubled, the areas are quadrupled in size, but their number is reduced to a quarter. Each result was upsampled with nearest-neighbour interpolation so that all are shown to be the same size. The hue encodes the angle of the bars, and the intensity encodes the intensity of detection.

Figure 6 shows the result of performing the method by Oliva-García et al. [33] at various scales on an area of an actual image containing barcodes and other patterns, such as separator lines or alphanumeric characters. Certain conclusions, which are helpful in proposing improvements in this initial method, can be drawn from it:

1. At smaller scales, the angle cannot be determined with high precision. However, at the smallest scale, it is observed that the method discriminates between vertical bars, in colours in the range of the blues, and horizontal bars, in colours in the range of the oranges.
2. If the scale is so small that only a uniform area is observed in a tile (e.g., the interiors of bars and spaces at scales of two and four), no activation is generated, which, although correct, represents a problem when grouping all the bars and spaces, with varying widths belonging to the same barcode.
3. On the other hand, at larger scales, there may be tiles that simultaneously observe part of the barcode and the background. This poses a problem in accurately determining the boundaries of the code.
4. In the previous case, i.e., tiles containing both bars and background, but also in the case of tiles that contain a zone with only a few thick bars, with respect to a tile covering a zone with thin bars, the activation intensity decreases. This explains the variability in detection intensity while keeping the hue—where the angle is encoded—uniform in areas with barcodes at the greatest scale.
5. At large scales, a drop in detection intensity can also occur when there are differently angled structures within a single tile. This is the case of zones with alphanumeric characters. In these cases, unlike the previous two, in smaller-scale sub-tiles, there may be angular disagreement, and there may even be greater intensity of detection in the parts than in the whole.

### 2.3. Initial Ideas for the New Methods

**Figure 7:**
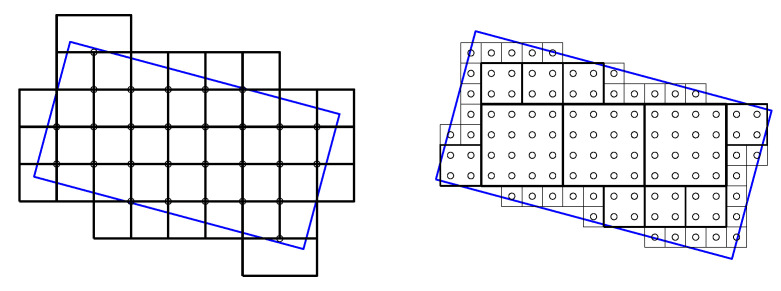
On the left, large tile size with smaller tile spacing, implying tile overlapping. On the right, the merging of tiles at various scales to optimise barcode coverage.

Figure 7 employs the symbology of Figure 5 to visually express the two variations that are proposed in this contribution.

The first idea is to decouple tile size and tile spacing, i.e., to allow overlapping among tiles. The subfigure on the left shows local evaluation with the largest tile size of Figure 5, but with tile spacing of half that size. It is outlined in Section 3 how to modify the stages prior to *t*, so that the latter can be optimally computed, allowing tile overlapping.

The second idea consists in the fusion of several scales so that smaller-scale tiles vote coordinately with larger-scale tiles in which they are framed, and vice versa. The solution has the spacing of the smaller scale, and the angular precision and accuracy of the larger scale. This achieves better definition of barcode edges and avoids, on the one hand, small-scale non-activation in uniform inner barcode areas and, on the other hand, disagreement voting at different scales in areas lacking barcodes.

It is shown that both variations increase the computation cost to different degrees, even if the computation of the previous stages, m<t, is always performed.

Moreover, ideas taken from the encoder–decoder architecture, proven to be successful in pixelwise segmentation tasks in the framework of **c**onvolutional **n**eural **n**etworks (CNNs) [22], are considered for the fusion of multiple scales but without resorting to machine learning. The encoder, in **a**rtificial **i**ntelligence (AI) terminology, is constituted, in this case, by the output of the DRT at all scales up to a certain tile_size. The mixing of the results is performed in the decoder, and as in AI, there are upsampling operations, but by remaining within the classical framework, there are no learnable weights. All of this leads to a scale-invariant method that can be executed in real time without latency.

This second method is presented in Section 4. In Section 5, details about optimised implementations for both methods are given. The paper concludes after presenting comparisons between these new methods and pre-existing ones in Section 6.

## 3. Overlapping Tiles for Increased Spatial Resolution

**Figure 8:**
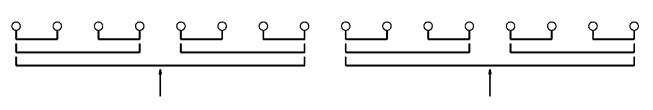
A depiction of how non-overlapping bands fuse at scales two and four to obtain a final output spacing of eight pixels per dimension. The depiction combines, in one picture, the evolution of vertical bands as they are shown at the bottom of Figure 4.

As explained in Figure 8, the unmodified DRT algorithm takes neighbour pixels in a vertical band consisting of pixels in horizontal positions $2\cdot k_{x}$ and $2\cdot k_{x}+1,\;\forall k_{x}\in \left\{0..N_{x}/2\right\}$, and computes and stores the sums with them with possible slopes −1,0, and +1. The next stage proceeds similarly and takes sums of length two from bands starting at $4\cdot k_{x}$ and $4\cdot k_{x}+2$ to create any possible sum of length four, in bands whose initial positions are now $4\cdot k_{x},\;\forall k_{x}\in \left\{0..N_{x}/4\right\}$. This is also illustrated in Figure 4. The final tile spacing for this example is eight, while the number of pixels is $N=N_{x}=N_{y}=16$. The algorithm that accomplishes such a task can be found in the work by Oliva-García et al. [33], and its computational complexity in big *O* notation is $O(N^{2}\log_{2}\left(\mathrm{tile}\_\mathrm{size}\right))$, as it stops after just a few stages. It is thoroughly discussed below in Section 3.1.

This algorithm must now be modified to allow overlapping, which implies computing, in stages greater than $s=\log_{2}\left(\mathrm{tile}\_\mathrm{spacing}\right)$, which are combinations that are not necessary in the original method. Specifically, where in the existing method, in stage *m*, computations have to be carried out with the separation of $2^{m}$, now, the separation must be $2^{\min(s,\;m)}$. This and the appropriate memory allocation for these new computations are the only aspects that need to be changed to make an output with that spacing in the upper stages possible.

**Figure 9:**
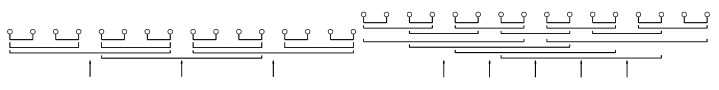
Illustration of how the final tile spacing determines the bands of computations in partial stages. Both represent N=16 and tile_size=8. They differ in the tile_spacing, which is 4 on the left and 2 on the right.

This is exemplified in Figure 9. On the left, it is shown what the spacing should look like for the same *N* and tile_size, but now with tile_spacing = 4. Nothing changes at scales m=1 and m=2, but something does at scale m=3. To introduce new line summations of length eight, no new combinations (with respect to the example in Figure 5) of length four and length two need to be created.

On the right side of the above figure, the case for tile_spacing =2 is shown, with *N* and tile_size remaining the same. In this case, additional bands must be created starting at scale m=2, because it is already greater than $\log_{2}\left(\mathrm{tile}\_\mathrm{spacing}\right)$. Note that the algorithm is still optimal in the sense that it only computes the necessary sums once, and if they are required again, it reuses them.

The new algorithm that allows overlapping, if tile_spacing differs from tile_size, is listed in Algorithm 1, highlighting the lines that need to be modified with respect to the algorithm by Oliva-García et al. [33]. If tile_spacing = tile_size, both algorithms perform the same computations and thus exhibit the same complexity.

### 3.1. Computational Complexity without Overlapping

The unmodified Radon transform [39] ends in stage $n=\log_{2}N$, with *N* being the side size of a square image, exhibiting computational complexity $O(N^{2}\log_{2}N)$ or, more precisely, examining the range of the for loops; the number of operations (two data reads, one addition and one write per iteration) for the horizontal partial DRT is

(1) $$4\sum_{m=1}^{n}\left(N\frac{N}{2^{m}}\left(2^{m+1}-1\right)\right)=4N^{2}\left(2\;n+\frac{1}{2^{n}}\right)\;\approx \;8N^{2}n.$$

To also consider the vertical lines, the same computations must be repeated after transposing the input, for a total of

(2) $$8N^{2}n+2N^{2}+8N^{2}n=N^{2}\left(16n+2\right).$$

In the method by Oliva-García et al. [33], on the other hand, a new parameter appears, tile_size, which indicates the desired size of local zones where to evaluate the existence of bars, i.e., the scale of the analysis. Let tile_size$\;=T=2^{t}<2^{n}$. Essentially, only the first *t* partial stages of the DRT are computed, discarding the results of the stages up to t−1. This step, as discussed above, requires $N^{2}\left(16t+2\right)$ operations.
**Algorithm 1** Computing the partial DRT of an image with tile overlapping.1:**function****partial_drt**(I,  tile_size,  tile_spacing)2:**Input:**I(x,y)⟶ Image consisting of N×N data3:**Input:**tile_size⟶ Size of the tiles4:**Input:**tile_spacing⟶ Spacing of the tiles5:**Output:**F(square,slope,displacement)⟶ Result of the transform6: 7:    $\mathrm{tile}\_\mathrm{size}\_\mathrm{bits}\leftarrow \lfloor \log_{2}\left(\mathrm{tile}\_\mathrm{size}\right)\rfloor$8:    $\mathrm{tile}\_\mathrm{spacing}\_\mathrm{bits}\leftarrow \lfloor \log_{2}\left(\mathrm{tile}\_\mathrm{spacing}\right)\rfloor$9:    N←size(I)[0]                                    ▹ Size supposed to be the same for x and y.10:    $F_{0}\leftarrow I$                                ▹ Initialise first stage with unmodified input.11:    **for** stage=1 to tile_size_bits**do**                     ▹ Allocate memory for next stages and fill with zeros.12:        [n_squares,n_slopes]←get_size_partial_drt(N,stage,tile_size,tile_spacing)13:        $F_{\mathrm{stage}}\leftarrow \mathrm{zeros}\left(n\_\mathrm{squares},n\_\mathrm{slopes},N\right)$14:    **end for**15:    **for** stage=0 to tile_size_bits−1**do**               ▹ Compute results in stage+1 from data in stage16:        $\mathrm{current}\_\mathrm{tile}\_\mathrm{size}\leftarrow 2^{\mathrm{stage}}$17:        $\mathrm{next}\_\mathrm{tile}\_\mathrm{size}\leftarrow 2^{\mathrm{stage}+1}$18:        [out_n_squares,out_n_slopes]←get_size_partial_drt(N,stage+1,tile_size,tile_spacing)19:        **for** out_y_square=0 to out_n_squares−1 **do**20:           **for** unsigned_slope=0 to out_n_slopes−1**do**                    ▹ slope as unsigned index21:               slope←unsigned_slope−next_tile_size+1               ▹ positive and negative slopes22:               ab_s←|slope|23:               s_sign←124:               **if** slope<0 **then**25:                   s_sign=−126:               **end if**27:               $s2\leftarrow \lfloor \frac{\mathrm{ab}\_s}{2}\rfloor$28:               rs←ab_s−2×s2                                ▹ remainder of absolute slope29:               slopeM←current_tile_size−1+s2×s_sign                    ▹ slopes of previous segments30:               incIndB←s_sign×(s2+rs)                    ▹ displacement among segments31:               **for** writeIdx=0 to N−1 **do**32:                   readIdx←writeIdx33:                   A←034:                   B←035:                   **if** 0≤readIdx<N **then**36:                       **if** stage<tile_spacing_bits **then**37:                          $A\leftarrow F_{\mathrm{stage}}\left(\mathrm{out}\_y\_\mathrm{square}\times 2,\mathrm{slopeM},\mathrm{readIdx}\right)$38:                       **else**39:                          $A\leftarrow F_{\mathrm{stage}}\left(\mathrm{out}\_y\_\mathrm{square},\mathrm{slopeM},\mathrm{readIdx}\right)$40:                       **end if**41:                   **end if**42:                   **if** 0≤readIdx+incIndB<N **then**43:                       **if** stage<tile_spacing_bits **then**44:                          $B\leftarrow F_{\mathrm{stage}}\left(\mathrm{out}\_y\_\mathrm{square}\times 2+1,\mathrm{slopeM},\mathrm{readIdx}+\mathrm{incIndB}\right)$45:                       **else**46:                          $B\leftarrow F_{\mathrm{stage}}\left(\mathrm{out}\_y\_\mathrm{square}+\mathrm{stage}-\mathrm{tile}\_\mathrm{spacing}\_\mathrm{bits}+1,\mathrm{slopeM},\mathrm{readIdx}+\mathrm{incIndB}\right)$47:                       **end if**48:                   **end if**49:                   $F_{\mathrm{stage}+1}\left(\mathrm{out}\_y\_\mathrm{square},\mathrm{unsigned}\_\mathrm{slope},\mathrm{writeIdx}\right)\leftarrow A+B$50:               **end for**51:           **end for**52:        **end for**53:    **end for**54:    **return** $F_{\mathrm{tile}\_\mathrm{size}\_\mathrm{bits}}$55:**end function**56:   57:**function**get_size_partial_drt(N,stage,tile_size,tile_spacing)58:    $\mathrm{stage}\_\mathrm{size}\leftarrow 2^{\mathrm{stage}}$59:    $n\_\mathrm{squares}\leftarrow \lfloor \frac{N-\min(\mathrm{stage}\_\mathrm{size},\mathrm{tile}\_\mathrm{size})}{\min(\mathrm{stage}\_\mathrm{size},\mathrm{tile}\_\mathrm{spacing})}\rfloor +1$60:    slope_size←2×stage_size−161:    **return** [n_squares,slope_size]62:**end function**

After the partial DRTs have been calculated, the variance in horizontal and vertical neighbourhoods of *T* values around the tile centres of bands in stage *t* are compared for 2T−1 angles. Those centres are located at positions $\left(k_{x}+\frac{1}{2}\cdot T,k_{y}+\frac{1}{2}\cdot T\right)$, with $k_{x}\in \left\{0..\frac{N_{x}}{T}-1\right\}$ and $k_{y}\in \left\{0..\frac{N_{y}}{T}-1\right\}$. This step increments the number of arithmetical and I/O data operations to $\left(2T-1\right)\frac{N_{x}}{T}\frac{N_{y}}{T}\left(2T+1\right)$. The algorithm that calculates the variance is listed as bar_detector by Oliva-García et al. [33] and is referenced with the same name in Section 4.

Typical values of *n* and *t* could be n=10 and N=1024, and t=5 and T=32, and in this case, the final number of operations represent just 48% of the operations required by the unmodified DRT reaching stage *n*. If, instead, the scale of analysis is T=16, with t=4, the number of operations would be 38%. This is due to the early halting of the transform, and as stated by Oliva-García et al. [33], some remarkably fast implementations can be obtained, even in mobile phones, when overlapping is not allowed.

### 3.2. Computational Complexity with Overlapping

Let tile_spacing$\;=S=2^{s}\leq 2^{t}<2^{n}$. Now, the number of operations of the partial DRTs, instead of $N^{2}\left(16t+2\right)$, increments to

(3) $$4\sum_{m=1}^{t}\left(N\frac{N}{\min(2^{m},2^{s})}\left(2^{m+1}-1\right)\right)=4N^{2}\left(\frac{3}{\min(2,2^{s})}+\frac{7}{\min(4,2^{s})}+\ldots +\frac{2^{t+1}-1}{min(2^{t},2^{s})}\right).$$

Afterwards, the variance comparisons add $\left(2T-1\right)\frac{N_{x}}{S}\frac{N_{y}}{S}\left(2T+1\right)$ operations.

In total, for N=1024 and T=32, but with S=2, the number of operations, instead of reducing, now increases by up to 9.27 times that of the unmodified DRT. For the case where S=4 and T=16, the increment is of just 1.13 times.

If comparisons are made not with the unmodified DRT but with the method by Oliva-García et al. [33], the proposed method is 19.19 and 2.94 times more costly, respectively, for the N,T, and *S* parameter combinations provided above. So this new method depends, to a large extent, on the output resolution and amount of overlapping set or, in other words, the quality improvement it provides. This encourages finding an alternative way to increase quality without such a steep increment in complexity, as discussed in Section 6.

In the following sections, what has been thus far called tile_spacing is now referred to as stride, as it is a more commonly used term in the CNN literature.

## 4. Multiscale Domain-Based Segmentation

### 4.1. Modifying DRT Output for Multiscale Analysis

In order to propose multiscale domain-based segmentation, the partial DRT algorithm was modified so that it returns not merely the last stage but all the $F_{i}$ initial stages, until it halts in the $\log_{2}\left(\mathrm{tile}\_\mathrm{size}\right)$ stage.

Now, it is possible to run bar_detector at all the scales, resulting in a pyramid of data detection actions. For the considered case, the sizes of the data structures are shown in Table 1 for input size 1024×1024 and tile_size=32.

These changes give place, optimally, to all the subfigures in Figure 6, simultaneously. The analysis of these data yielded interesting insights, as discussed in Section 2.2.1.

### 4.2. Finding Similarities with Machine Learning-Based Algorithms

**Figure 10:**
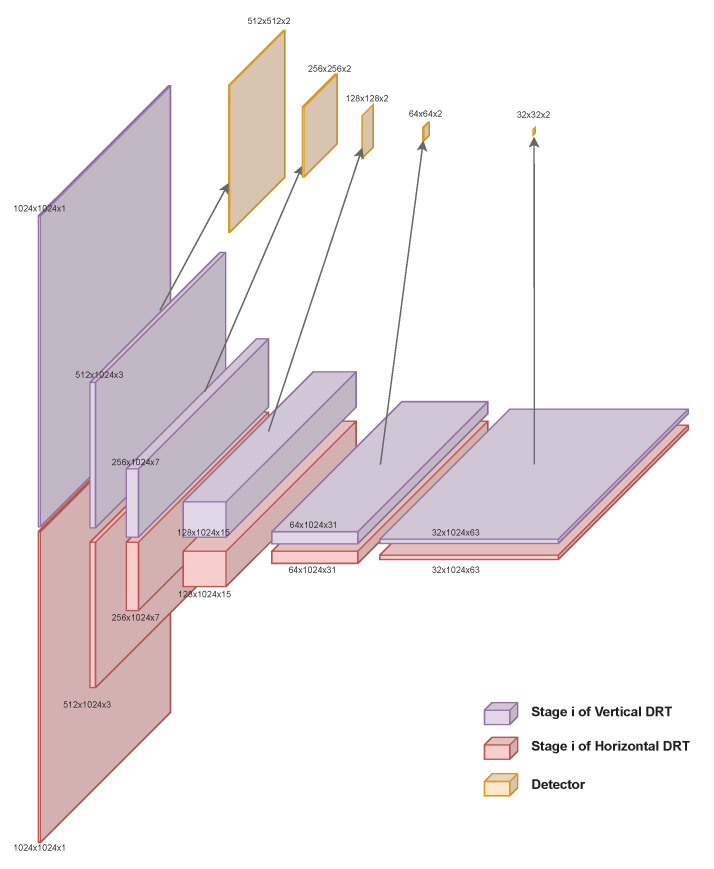
Architecture of the detector based on the partial DRT, as described in [33] but taking advantage of all the calculated stages up to tile_size=32.

Taking advantage of the goodness of each scale is the goal of this approach. There are barcodes that can be correctly detected at different scales, mainly because of the size of the barcode but also because of lens blur and motion blur. By looking at the pattern of the data structures that appear, where we have maps at different scales and the spatial resolution becomes smaller, while the angle-domain resolution becomes bigger with the scale, it almost resembles a CNN for classification. The depth, channels, features, or classes of a classification network are equivalent to the angles in our problem. For example, the new pyramid of detection data, shown in Figure 10, is remarkably similar to the architecture of the VGG16 model [40] widely used in the literature, shown in Figure 1.

Models such as VGG16, LeNet-5 [41], and GoogleLeNet [42] all share the same purpose: the classification of objects that are represented in an image. They also have the same funnel shape, which starts with the size of the image and reduces the spatial dimensions while increasing the depth dimension—the number of classes for classification. In the partial DRT-based detector, the same process happens, substituting the concept of class with that of angle. This “funnel” shape is what constitutes an encoder.

### 4.3. Adapting the Existing Algorithm to Resemble an Encoder

**Figure 11:**
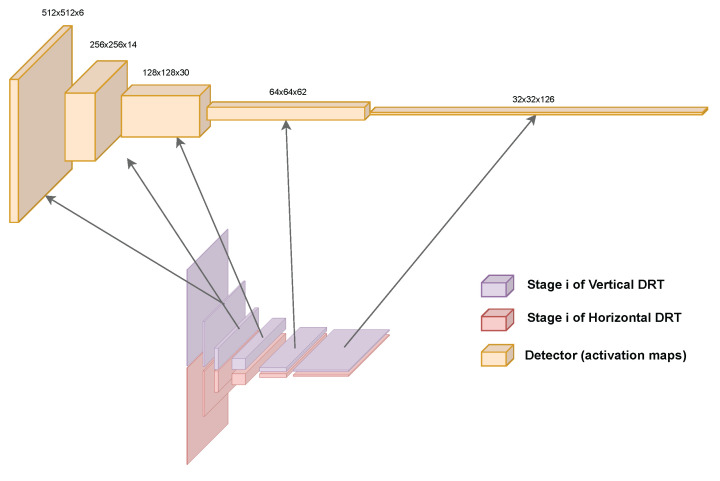
New encoder topology, based on the work by Oliva-García et al. [33] and modified as described in this section. Note that for simplicity, the partial DRT data structure was made smaller in scale for display purposes.

Returning to the problem at hand and reformulating some of the vocabulary used, it can be said that in [33], there is already an algorithm that can classify the bars present in the input image into angles. That is, a probability of each of the 126 angles is obtained for each of the blocks that define the spatial dimensions in the last stage (32×32). In the original bar_detector algorithm, the probability of each angle is discarded, and only the maximum angle is written into the output. To have an algorithm somewhat equivalent to the encoder of a CNN and avoid losing data, this was changed so that all the probabilities can be obtained. This change can be observed in the modified version of Algorithm 2 and gives place to the topology that is shown in Figure 11. The complexity of the algorithm described in [33] remains untouched, although the amount of memory that is needed increases.
**Algorithm 2** Creating the activation maps for the CNN-like encoder from partial DRTs.1:**function** **BAR_DETECTOR**(H, V)2:    **Input:** H(x,y)⟶ Horizontal partial DRT of size [n_squares,n_slopes,N]3:    **Input:** V(x,y)⟶ Vertical partial DRT of size [n_squares,n_slopes,N]4:    **Output:** activation_map(x,y,slope)⟶ Per tile and slope activation map5:    n_squares←size(V)[0]               ▹ n_squares is N over tile_size6:    n_slopes←size(V)[1]                    ▹ n_slopes is 2xtile_size - 17:    N←size(V)[2]8:    activation_map←zeros(n_squares,n_squares,n_slopes×2)9:    $tile\_size\leftarrow \frac{N}{n\_squares}$10:    **for**    x_square=0   to   n_squares−1 **do**11:        **for**    y_square=0   to   n_squares−1 **do**12:           $x\_central\leftarrow x\_square\times tile\_size+\frac{tile\_size}{2}$13:           $y\_central\leftarrow y\_square\times tile\_size+\frac{tile\_size}{2}$14:           **for**    slope=−tile_size+1   to   tile_size **do**15:               $start\_h\leftarrow y\_central-\frac{tile\_size}{2}-\frac{slope}{2}$16:               $end\_h\leftarrow y\_central+\frac{tile\_size}{2}-\frac{slope}{2}$17:               values_h←H(x_square,slope+tile_size−1,start_h:end_h)18:               $start\_v\leftarrow x\_central-\frac{tile\_size}{2}+\frac{slope}{2}$19:               $end\_v\leftarrow x\_central+\frac{tile\_size}{2}+\frac{slope}{2}$20:               values_v←V(y_square,−slope+tile_size−1,start_v:end_v)21:               $value\_horizontal\leftarrow \bar{|values\_h-\bar{values\_h}|}$22:               $value\_vertical\leftarrow \bar{|values\_v-\bar{values\_v}|}$23:               v←|value_horizontal−value_vertical|24:               **if** value_horizontal>value_vertical **then**25:                   activation_map(x_square,y_square,slope+tile_size−1)←v26:                   activation_map(x_square,y_square,slope+tile_size−1+n_slopes)←−v27:               **else**28:                   activation_map(x_square,y_square,slope+tile_size−1)←−v29:                   activation_map(x_square,y_square,slope+tile_size−1+n_slopes)←v30:               **end if**31:           **end for**32:        **end for**33:    **end for**34:    **return**   activation_map35:**end function**36:   37:**function** **ENCODER**(I)38:    **Input:** I(x,y)⟶ Image consisting of N×N data39:    **Output:** activation_maps(scale,x,y,angle)40:    tile_size←32                          ▹ Suitable for image size of 1024.41:    tile_spacing←tile_size     ▹ Notice that tile_spacing=tile_size. Fusion works without overlapping.42:    hdrt←partial_drt(I,tile_size,tile_spacing)43:    $vdrt\leftarrow partial\_drt(I^{T},\;tile\_size,\;tile\_spacing)$                  ▹ Notice the transposed input.44:    **for**    stage=0    to    $log_{2}\left(32\right)$ **do**45:        activation_maps(stage)←bar_detector(hdrt(stage),vdrt(stage))46:    **end for**47:    **return**   activation_maps48:**end function**

### 4.4. Testing the Encoder

**Figure 12:**
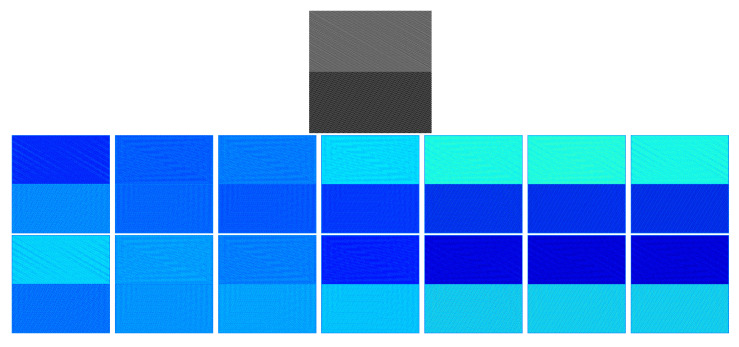
From **left** to **right**: input image and activation maps at scale = 2, slicing for slopes 0 to 12, which correspond to angles in the range [−π/4,3π/4]. A colormap is used to represent intensity, ranging from a darker blue to a lighter blue. The upper part of the image contains lines at an angle of π/6, and the lower part of the image contains lines at an angle of π/3. Note that the activation map correctly activates more in the corresponding slice of each half.

**Figure 13:**
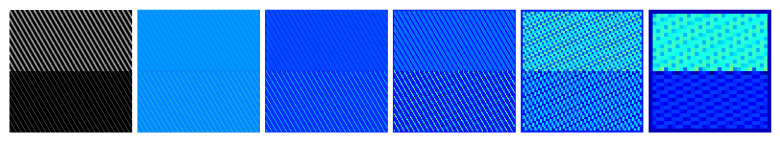
From **left** to **right**: input image and activation maps for the central angle of each scale, from finer (first) to coarser (last) scales. A colormap is used to represent intensity, ranging from a darker blue to a lighter blue. It is clear that finer lines (lower part) produce more intensity at the second and third scales, while coarser lines (upper part) are better detected at the last scale.

With these simple adaptations, the authors have described a topology that is similar to those of the encoders of the mentioned CNNs, and the activation maps can now be studied. In addition, this encoder is optimal, because it is based on the DRT [36], which is optimal in itself. If the maps are analysed by choosing a scale and comparing all the 2D activation maps for all the angles, the pixels should gain intensity whenever a bar oriented along the angle of the slice is present. On the other hand, if an angle is chosen, and the scale is traversed, the pixels should gain intensity whenever a bar has the adequate size to be detected on the scale of the slice. To corroborate the first experiment, a synthetic image was prepared. This image was divided along the vertical axis such that the upper part contained lines at an angle that was different from that in the lower part. Therefore, the 14 activation maps at the second scale, of size 256×256, had to gain intensity in different activation map angle slices, indicating the angle of the lines; see Figure 12. For the second experiment, a synthetic image was prepared, again dividing it into two halves, where the upper part contained coarser lines and the lower part contained finer lines. This time, the angle index was fixed to match the angle of the lines, and the scale domain was traversed. The five activation maps had to gain intensity at different times, meaning that the lines were better detected at different scales, as demonstrated in Figure 13.

### 4.5. Designing a Decoder

Now that the encoder has been designed and tested, a decoder is needed to perform pixelwise segmentation. As shown above, in Figure 2, a way of taking advantage of an encoder to perform segmentation is to build a topology that is structured similarly to a mirror. Starting from the last operation of the decoder, that is, the coarser scale, for each operation of the encoder that lowers the spatial resolution, downsampling, a new operation that does the opposite, increasing the resolution, is placed, recreating the same structure but in reverse.

**Figure 14:**
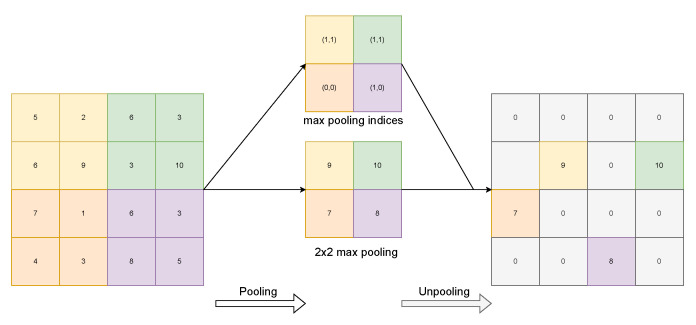
Example of unpooling operation. The pooling operation reduces dimensionality, and the unpooling operation increases it. Note that there are “gaps” where no indices refer to the array on the right.

In the case of VGG16, the downsampling operation is a max pooling operation. This operation consists in reducing the dimensionality of a layer by choosing the maxima of the features contained in the sub-regions of the biggest domain. SegNet uses an unpooling operation, which uses the indices (the arrows in Figure 2) of the pooling operation for placing the features at the appropriate locations of the biggest domain; see Figure 14.

In this way, the features detected in the activation maps at a coarser scale are translated into a finer scale using the indices of the mirror pool operation on the encoder part, so compared with upsampling based on bilinear interpolation, the features are located more precisely. This allows one to retrieve the spatial resolution that is lost in the encoding part. As we can see in Figure 14, there are gaps, parts of the map where there are zeros, because no index refers to those locations. This information is filled in by layers that follow the unpooling operation. These layers are learned. The authors of SegNet avoided implementing a learnable upsampling operation in order to speed up training and inference by letting the convolutions and activation functions perform that process. Other models use learned upsampling operations such as transposed convolution, also called up-convolution or even (confusingly) deconvolution. This is the approach that the **f**ully **c**onvolutional **n**etwork (FCN) [43] model uses. This operation does not produce “gaps” as the unpooling operation does, and interestingly, the activation maps resulting from upsampling are summed with the ones of the corresponding downsampling operation.

Regardless, in our approach, both upsampling approaches have the same characteristics, i.e., there is a learnable part, and the operations that achieve the process are just pooling operations, convolutions, and activation functions. Pooling and convolutions that have strides can achieve the same objective, but a pooling–unpooling configuration always needs to be matched with learnable convolution in order to solve the “gaps”. Of course, this DRT-based encoder is not similar to a CNN encoder, as classification and operations such as max pooling do not exist. Therefore, a bit of imagination is needed to create an operation that is opposed to the downsampling operation and understand what the role of the learnable part (the convolutions) is. Taking both analysed pixelwise segmentation models, a decoder was designed.

#### An Upsampling Operation for a DRT-Based Encoder

First of all, the first building block is the upsampling operation. Since there is no learnable part, this should be straightforward. Taking inspiration from unpooling, the goal is to generate a set of activation maps that have doubled their dimensions in width and height. So, at a scale that has size h×w×c, the output is 2·h×2·w×c. In order to find the indices of the non-existing pooling operation, the arguments of the maxima of the activation functions at the next finer scale are used. Since this scale has a smaller angle dimension, this simple formula is used to calculate the finer angle index, $f_{a}$, using the coarser angle index, $f_{c}$, where *C* is the size of the angle domain at the finer scale.

(4) $$f_{a}=\left\lfloor \frac{f_{c}}{2}\right\rfloor \;\mathrm{mod}\;C$$

**Figure 15:**
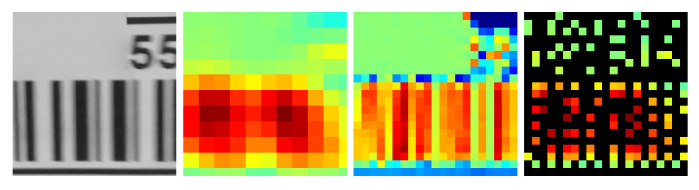
From **left** to **right**: cropped part of input image; coarse activation map for horizontal angles; fine activation map for horizontal angles; the result of performing the unpooling described in Algorithm 3. Note that there are “gaps” and that each activation function that got propagated used the coarse angle information, and the correct spot was calculated by finding the argument of the maxima within the corresponding area at a finer scale.

Algorithm 3 implements the custom unpooling operation for complementing the DRT-based encoder. There is an argmax operation in line 7. This means that the function is already different from the normal unpooling that one finds in a machine learning frameworks such as tensorflow or pytorch; only the max angle gets propagated. This design choice is because of two reasons. One of them is that this acts as an activation function, so not all the features propagate, and a priority is implemented. The other one is that while it is true that to resemble an actual unpooling operation, all the angles should propagate and, later on, an activation function should prioritise, this work is largely focused on speed, which allows us to significantly reduce the number of operations. Therefore, with this algorithm, the activation function and the unpooling behaviours of a decoder such as the one in SegNet are solved. Figure 15 shows the effect of executing this algorithm. As expected, the feature propagates to the right spots, but there are gaps. The improvement in accuracy of each of the activation functions that get propagated at the different scales is what makes it possible to gain precision in the final pixelwise segmentation.
**Algorithm 3** Unpooling operation for a DRT-based encoder.**Input:**F(x,y,c)⟶ Fine activations of size [n_squares·2,n_squares·2,n_angles_fine]**Input:**C(x,y,c)⟶ Coarse activations of size [n_squares,n_squares,n_angles_coarse]**Output:**N(x,y,c)⟶ New activations of size [n_squares·2,n_squares·2,n_angles_coarse]1:**function** **UNPOOL**(C, F)2:    n_squares←size(C)[0]3:    n_angles_fine←size(F)[2]4:    n_angles_coarse←size(C)[2]5:    **for**    i=0   to   n_squares−1 **do**6:        **for**    j=0   to   n_squares−1 **do**7:           max_angle←argmax(C(x,y,:))8:           $max\_angle\_finer\leftarrow \left\lfloor \frac{max\_angle}{2}\right\rfloor \;\mathrm{mod}n\_\mathrm{angles}\_\mathrm{fine}$
9:           max_val←0;vj←−1;vi←−110:             forii=0to2do11:                forjj=0to2do12:v←F(i·2+ii,j·2+jj,max_angle_finer)13:ifv>max_valthen14:max_val←v;vi←ii;vj←jj15:endif16:endfor17:endfor18:N(i·2+vi,j·2+vj,max_angle)←C(i,j,max_angle)19:endfor20:endfor21:returnN22:endfunction

Finally, the hardest part to design is the one that, in models such as FCN or SegNet, is learnable. The DRT-based encoder should mimic what networks learn to do in their smart upsampling. The learnable part of a decoder performs three actions:

1. Propagating features that are prominent at a coarser scale into a finer scale. This is achieved with the unpooling in Algorithm 3.
2. Propagating features to the neighbouring vicinity. In this problem, vicinity is defined in the *x*- and *y*-dimensions but also in the angle (depth) dimension.
3. Allowing features that are prominent at any scale to be considered. As Figure 13 suggests, there can be bars that are only detected at finer scales, so coarser detection should not overthrow these if they are more prominent.

So, two aspects have yet to be solved. Considering that the remaining palette of operations that a CNN uses are convolutions and activation functions, it is easy to conclude that the propagation of features at the same scale to the neighbouring vicinity can be achieved with a low-pass filter. So, this was solved as follows: a 9×9 separable Gaussian filter is applied to each activation map for each angle; then, the angle dimension is also filtered but only with a size of three.

**Figure 16:**
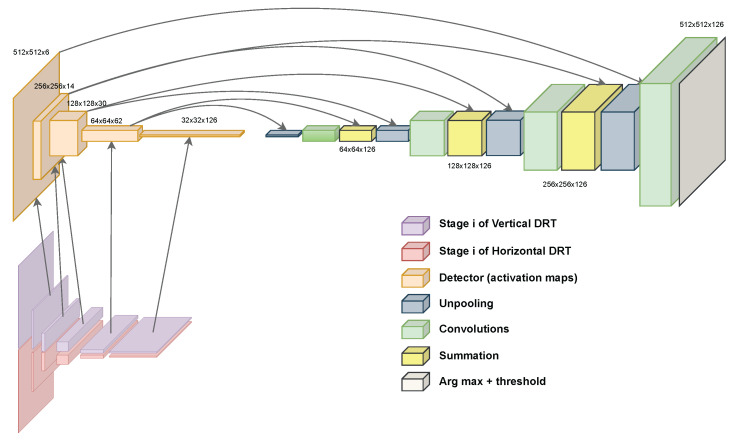
Full encoder–decoder architecture, where the DRT-based encoder is matched with the newly designed decoder integrating the operations described in this section.

Finally, to allow features to appear in the middle of the decoding stage, at an intermediate scale, the result of the activation maps of the encoder are summed into the new activation maps, similarly to the FCN. This is a simple pixelwise sum operation, and no factors are applied. In this way, a decoder algorithm was obtained, and together with the encoder, a pixelwise segmentation algorithm for finding barcodes was designed. The final architecture of the segmentation algorithm is shown in Figure 16.

## 5. Implementation

With the goal of real-time barcode detection for augmented reality, two different programming environments were chosen. The first one, used for designing and debugging algorithms, was Python [44], which brings easiness of use and a very low development cost. This makes the fast prototyping of new ideas very affordable. The downside of using Python for these kind of computer vision tasks, given its interpreted nature, is that sometimes, a bottleneck can impede the testing of some ideas, because they are too computationally heavy. For mitigating this, to some extent, the authors used the Python package Numba [45] as an accelerator. The version used for this work was Python 3.10.

The second environment was chosen to make lower-level, accelerated implementation possible, which is suitable for targeting computing units such as CPUs, GPUs, and DSPs, and platforms such as desktop, mobile, and web. It consisted of code written in C++17 [46]. The algorithms were written in the Halide language [47]. Halide is a programming language embedded in C++ that decouples the declaration of computer vision algorithms from the schedules. This allows one to easily try different scheduling strategies and writing different schedules for different architectures. It also provides different auto-schedulers [48,49,50] that automatically generate schedules that can be used as a starting point or even become the final schedule. In this case, the authors used the Adams2019 [48] auto-scheduler for generating all the needed schedules. From the possible targets, the authors chose to test the results and execution time on a desktop CPU (Intel i9-9900) and a mobile phone CPU (Qualcomm Snapdragon 888).

Both Python and C++ versions are available from the GitHub repository [51].

### 5.1. Pruning and Optimisation

Three main types of optimisation were carried out before any hardware-specific acceleration. From now on, the authors refer to four algorithms by their acronyms:

- PDRT 2: The original algorithm of the partial discrete Radon transform as described in [33], executed with tile_size=2. This means that a single step of the DRT was calculated.
- PDRT 32: The original algorithm of the partial discrete Radon transform as described in [33], executed with tile_size=32. This means that five steps of the DRT were calculated.
- PS DRT: Partially strided DRT-based detector as described in Section 3. The chosen parameters were tile_size=32 and stride=2.
- MDD DRT: Multiscale domain detector based on the DRT, as described in Section 4.

The average absolute deviation (D) was calculated for each tile and each slope; see lines 21 and 22 of Algorithm 2. This can be better expressed as

(5) $$\bar{A}=\frac{1}{n}\sum_{i=1}^{n}x_{i}$$

(6) $$D=\frac{1}{n}\sum_{i=1}^{n}|x_{i}-\bar{A}|$$

Considering the architecture of modern computing units and the goal of these operations, the authors realised that this is wasteful and can be substituted with the following, with no impact on quality:

(7) $$\tilde{D}=\sum_{i=1}^{n-1}|x_{i}-x_{i+1}|$$

This approximation was already considered in [33].

To achieve an implementation that takes advantage of the fact that the discrete Radon transform works with only integer arithmetic, the data type chosen for the buffers was 16-bit integers, which is enough to hold the range of the sums that take place in the execution of a DRT for an image of 1024×1024 and for a tile_size=32 (five stages). Because the PS DRT and MDD DRT algorithms calculate differences and thus hold negative values in some variables, the signed 16-bit integer data type was chosen instead of its unsigned variant. This speeds up computation when compared with the 32-bit floating point data type that was used when designing the algorithm, and the results are equivalent. This approach was also implemented with PDRT 2 and PDRT 32.

The resulting number of angles in this algorithm is excessive since such fine-grained precision on the angle is not needed for this task. By profiling the execution of the MDD DRT algorithm, it can be noticed that about 80% of the time is spent on the last convolution block. That is because the domain over which the convolution is executed is 512×512×126. By halving the size of the output angle dimension in each unpool until it reaches size 30, the final quality remains almost equal, and the number of operations is greatly reduced. The sizes of the operations of the final algorithm following optimisation are shown in Table 2.

### 5.2. Choosing a Fast Approximation of a Gaussian Filter

In Section 4, low-pass filtering is described as a 9×9 separable Gaussian filter. There are many works that deal with approximations of low-pass filtering that use different strategies, such as approximating a Gaussian filter using a box filter with multiple passes [52], using a discrete kernel [53], or performing filtering in a downsampled image [54]. Having implemented all the operations in integer arithmetic, it made sense to approximate the Gaussian filter using a set of two box filters of size 3×3, which were again implemented in a separable fashion. This, as the simplest option, gave a good result in terms of speed and quality.

## 6. Results and Conclusions

### 6.1. Time Results

**Figure 17:**
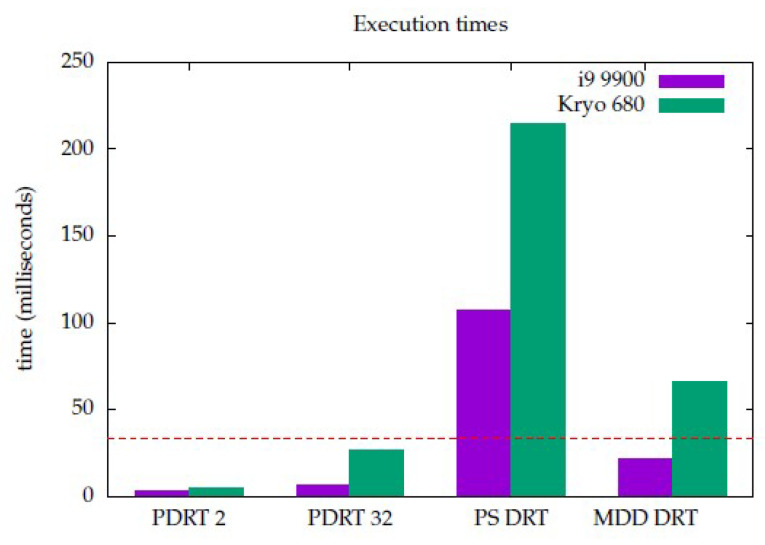
Average execution times of the Halide implementation of the four algorithms. These measurements were performed on an i9 9900 desktop CPU and on the CPU of the Qualcomm Snapdragon 888 mobile SoC. The red dashed line emphasises the 1/30 s time limit.

The results of benchmark execution—with enough repetitions to ensure repeatability—are shown in Figure 17. These correspond to the auto-scheduling of the Halide/C++ implementation. Python time results were not measured, as its implementation purpose was solely that of prototyping and designing. Two targets were chosen as a test bed. One was the Intel i9 9900 desktop processor, with eight cores @ 3.10 GHz, and the other was a state-of-the-art CPU of a mobile **s**ystem **o**n a **c**hip (SoC), **S**nap**d**ragon (SD) 888, which has eight Kryo 680 cores @ 3 GHz. The latter was chosen because most AR applications run on a mobile device. The GPU of the same SoC could have also been an interesting target and will be addressed in future work.

PDRT 2’s and PDRT 32’s average execution time was far below the 33 ms threshold. Nevertheless, as stated in Section 2.2.1, these algorithms were not the focus of this work, as their output is not appropriate for segmentation, and are reported here as a mere reference. PS DRT, which, qualitatively, had acceptable segmentation results, was very far from a real-time, AR-ready implementation, since its execution took 107 and 215 milliseconds on i9 and SD, respectively. Lastly, MDD DRT, which had good segmentation results, achieved real-time execution times below 33 ms (21 ms) on the desktop CPU but not on the mobile CPU (66 ms).

### 6.2. Runtime Comparison

In order to be able to draw conclusions from directly comparing our methods with those by other authors, the conditions had to be similar, but previous methods mostly use low-resolution inputs and ignore mobile platforms.

It can be seen in Table 3 that our MDD DRT method was faster than any other method when run on a CPU, even if it required the highest input resolution. Moreover, it was the only one performing under the 33 ms time limit. Neural network-based methods were represented by the hand-crafted method by Zharkov and Zagaynov [17] and by our measurement of inference times of YOLO v5 small, as it was selected by Kamnardsiri et al. [24] as a good representative of the state-of-the-art methods in terms of both accuracy and speed.

Previous methods that manage to get below the 33 ms threshold are able to accomplish this because they target desktop GPUs, which this paper does not consider, as they are not a realistic platform for wearable AR devices.

Although it can be claimed that our methods outperformed the literature alternatives, our challenge was achieving execution within self-imposed time constraints without neglecting the quality requirements, and in this sense, the second proposed method can be considered to be fully satisfactory.

### 6.3. Accuracy Comparison

**Figure 18:**
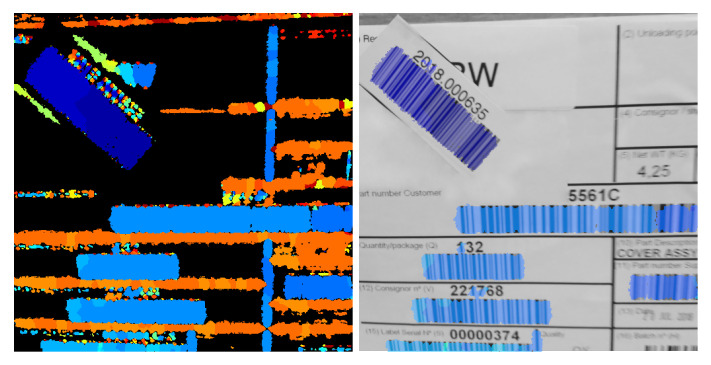
**Left**: unprocessed output of the MDD DRT segmenter. **Right**: superimposed on the input image, connected regions whose aspect ratio between the major and minor oriented axes is consistent with a barcode and whose area exceeds a minimum threshold.

It should be noted that the output of our algorithms, i.e., the classification of areas according to bar presence and orientation, cannot be directly used as a barcode detector. In this sense, the outputs contain a lot of false positives, that is, areas where the segmenter detects potential barcodes, but where there are none. Since angular information on the orientation of the codes is available, a simple criterion of oriented eccentricity in connected regions is adequate to eliminate most of them. This simple criterion—which probably has a good margin of improvement—is the one used to turn our segmentation outputs into masks of detection for subsequent accuracy comparison. A particular example is provided, for a cluttered scenario, in Figure 18.

#### Accuracy Metric and Benchmarking Datasets

Given a binary mask *G* for ground truth and a binary mask *F* for the detection results, a commonly accepted metric is the **i**ntersection **o**ver **u**nion (IoU) or Jaccard index, defined as

(8) $$J\left(G,F\right)=\frac{\left|G\cap F\right|}{\left|G\cup F\right|}$$

The overall detection rate, *D*, for a given Jaccard threshold *T* is defined as the ratio of images in a dataset *S* where the Jaccard index is greater than that threshold.

(9) $$D_{T}=\frac{\left.\sum_{i\in S}J\left(G,F\right)\geq T\right)}{\left|S\right|}$$

The averaged detection rate for several thresholds can be obtained as

(10) $$D_{T1-T2}=\frac{\sum_{T\in T1}^{T2}D_{T}}{T_{2}-T_{1}}$$

To compare our methods in terms of accuracy, the detection rates on two datasets for which binary ground truth masks are available were calculated. The first dataset (Zamberletti et al. [16]), named “Arte-lab Rotated”, contains 365 images of sizes 640 × 480 and 648 × 488. The second one (Wachenfeld et al. [4]), named “WWU Münster”, contains 1055 images of size 2592 × 1944. Both datasets were labelled by Zamberletti et al. [16] with binary masks that indicate what pixels of an image correspond to a barcode. These masks are of sizes 640 × 480 and 648 × 488, so this was the resolution used in the tests. There are 595 masks for WWW Münster, and from the 365 masks of Arte-Lab Rotated, 5 were discarded due to them being wrong. Our method would have benefited if these inputs had had 1024 × 1024 resolution. As that was not the case, we preferred to resize them to the expected size by adding zeroes to the borders, instead of upsampling them.

Metrics for both methods are included in Table 4. PS DRT was not only the slowest but also the one that profiled the barcodes the worst. Therefore, it is not the focus of attention in the following.

Looking at the results obtained with MDD DRT, it is worth noting that it improved upon some classical methods, obtaining detection rates of 90 and 95 % at 0.5, but it remained below the perfect detection rate at this threshold, which the majority of the methods based on neural networks and the classical method in [11] achieved.

The limits of the proposed method are discussed in Section 6.5; nonetheless, it is worth commenting here that some defocused barcodes that can be easily detected with state-of-the-art methods were not recognised by ours. This can be explained by the low resolution and because they presented compression. Compression artefacts make fine-grained activation maps differ in angle compared with the coarse-grained ones. This makes our method consider the detection unreliable and induces low scores even at $D_{0.5}$.

Nonetheless, the results obtained on these datasets, considering the information in Table 3, can be considered successful, given that the proposed method is designed for inputs that do not contain artefacts and are of greater resolution. This is discussed further in Section 6.5. On the Münster dataset, which is also compressed but was downsampled from higher-resolution inputs, the results of the proposed method were better, reaching 95% detection accuracy and superseding several methods in the $D_{0.5-0.9}$ metric, because it profiled barcodes that can be troublesome for other methods quite well.

### 6.4. Synthetic Parametric Tests

**Figure 19:**
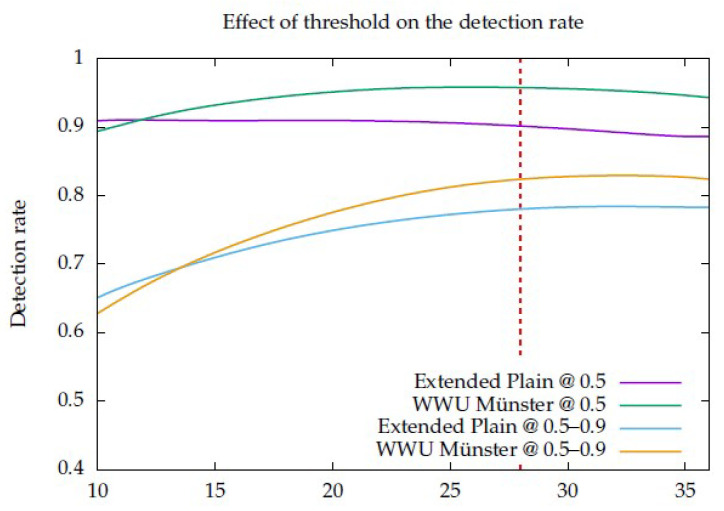
The MDD DRT algorithm was executed for thresholds ranging from 10 to 38 on both the WWU Münster dataset and the Arte-lab Rotated dataset. The selected threshold value is marked with a dashed red line.

The only one of the parameters of the MDD DRT algorithm that can be considered free is the activation threshold. For this single parameter, the evolution graphs of the accuracy metrics are presented for the two datasets in Figure 19. Given that the two datasets are different, a threshold of 28 was selected to achieve a good compromise between the two.

**Figure 20:**
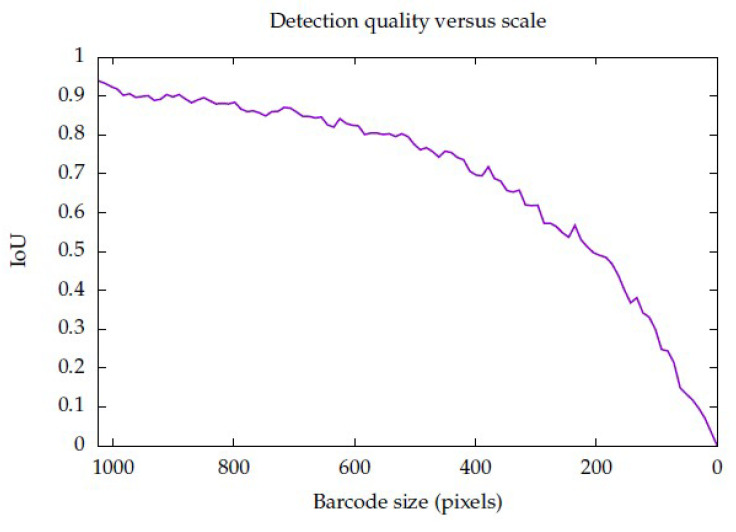
The IoU of a barcode that was scaled to different sizes.

It is interesting to evaluate how the IoU metric changes with the size of a barcode. For this, the following experiment was carried out: An image containing a barcode was synthesised in such a way that the size of the pixels of the barcode was programmatically set, ranging from 1024—the maximum size possible if the barcode is horizontal—to 0, varying at intervals of 10.24 pixels, such that 100 measurements were performed. The ground truth mask was synthesised at the same time. The MDD DRT algorithm worked as expected, and the quality of detection slowly degraded with the size of the barcode. The result of such analysis can be seen in Figure 20.

**Figure 21:**
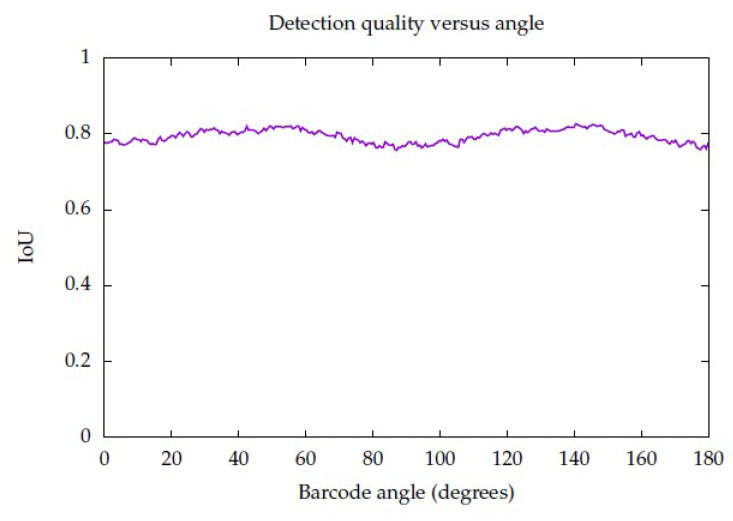
The IoU of a barcode that was rotated to angles ranging from 0 to 180 degrees.

The angle is a factor that needs to be evaluated, since the MDD DRT algorithm should provide angle invariance. A new experiment was devised: The same barcode generation scheme used in the scale experiment was used; in this case, the size was fixed at 512 pixels in width, and the angle was varied from 0 to 180 degrees at intervals of 0.6, so that 300 images were evaluated. The behaviour was as expected: the detector performed equally well at all angles. The result of this experiment can be appreciated in Figure 21.

### 6.5. Qualitative Analysis

**Figure 22:**
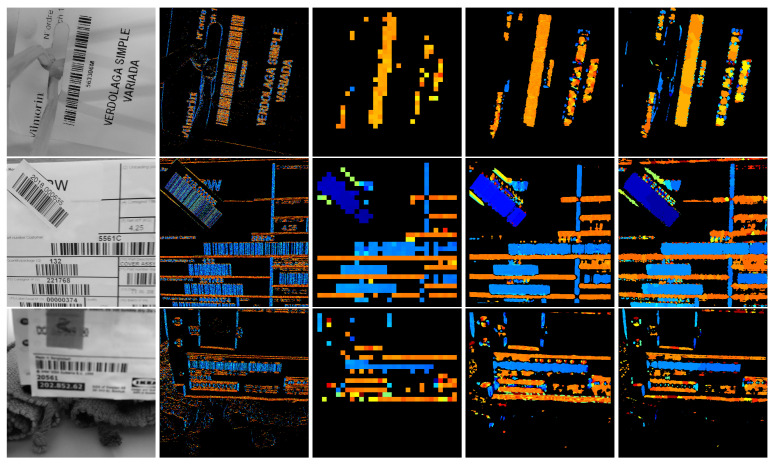

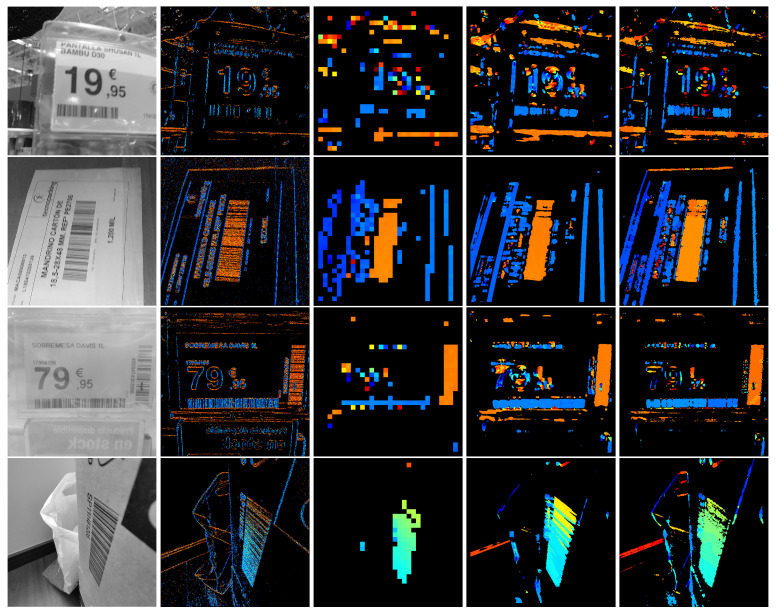
Columns, from left to right: input image and output of algorithms PDRT 2, PDRT 32, PS DRT, and MDD DRT. Rows, challenging scenarios, from top to bottom: elongated, cluttered, mild lens blur, strong lens blur, motion blur, low contrast, and shear.

Well-defined, sharp and large codes are not a challenge for previous proposals, nor for the current one. Hence, a qualitative discussion on more challenging situations, for which representative samples are shown in Figure 22, was carried out. It is recommended to view the samples enlarged in the digital version of the paper. The same examples were analysed with the existing Radon-based bar detector method at two fixed scales and with the two new methods, i.e., partially strided and multiscale domain detection.

The discussion on the samples is as follows:

- The first row shows a barcode that could be considered unproblematic. Although it contains some contrast variation due to paper bending and quite separated bars, its main difficulty, only for some neural network-based methods, is that its aspect ratio is approximately 1:8. This was not a problem for the PS DRT algorithm, nor for MDD DRT. The fixed-scale algorithms, on the other hand, had problems in properly contouring the code boundaries. This was consistently found in the rest of the examples; therefore, these algorithms, whose outputs are of interest mainly because they are the base and apex of the data pyramid on which the PS DRT and MDD DRT methods operate, are not further discussed.
- The second row shows an image containing a multitude of codes. Some are out of focus; some are rotated; and several dividing lines and alphabetic characters make it difficult to separate them correctly. In addition, a couple of codes are incomplete at the lower end. The MDD DRT method contoured the codes better than the PS DRT method. It was also more sensitive to the angle of the lines and tended to make them more uniform, but this does not result in definite advantages in a real-world application. Instead, as seen in the previous subsection, the focus from now on is exclusively on the MDD DRT method, because the PS DRT method simply does not fit into the time constraints. The segmentation was accurate, and even the small and trimmed code on the border was detected.
  The main disadvantage of the MDD DRT method is that it joins characters close to a barcode if they are made up of strokes with the same slope. This is the case for characters such as 0, 1, 7, O, I, etc. Fortunately, these “overflows” can be counteracted in the post-processing phase, which is not covered in this article.
  Bars or outlines close and parallel to the ends of the codes are also problematic, since the measures taken to save the internal areas with low texture but belonging to the codes force their undesired inclusion.
  This image is the same as the one used in Figure 18 to illustrate how to get rid of false positives with a simple criterion.
- The third and fourth rows illustrate the cases of barcodes affected by strong and extreme lens blur, respectively. The proposed methods still managed to adequately contour the input of the third row but not that of the fourth row. The final scale of the algorithm, with analysis zones of 32 × 32 pixels, allowed bars to be detected, even though, at a smaller scale, the blur merged them with other bars. Also depending on the loss of contrast, at some point, the detector stops triggering. In an example such as this, the detector showed its performance limits, but regardless of whether it can be further improved as a future line of work, it is already a major advance over previous methods, which simply do not work at all when facing heavily defocused barcodes.
- The next row presents a case of severe motion blur in the direction that affects the code bars the most. Both types of blur, lens blur and motion blur, were treated in the same way, and in cases such as this, the detector behaved as expected.
- The image in the sixth row combines very low contrast with glare and smudges on the codes. As it can be seen, the detection is satisfactory, but in this extreme case, the combined effect of a wide bar, low contrast, and glares oriented in the opposite direction with respect to the bars broke the code into two disconnected regions. This is another limitation of our method. In PS DRT, this does not occur, since it is the voting scheme in MDD DRT that penalises character regions with sharp slope changes nearby that works against the correct detection. Fortunately, in the rare occasions in which this happens, it can be solved in post-processing, since angle labels per region are available.
- The last example is a case of extreme perspective that makes the observed slope of the bars of the same code vary. The MDD DRT method solved it very well, and the bars changed smoothly so that they could be grouped in a single region.
  The problems of joining nearby characters are once again evident.
  The lack of precision of the boundaries in the upper-right corner of the code is due to another factor. Unlike the other sample images in this figure, which were taken by directly accessing the camera, i.e., without compression and disabling edge enhancements, in this case, it is a JPEG image where there are compression artefacts. Those artefacts, at a low scale, make the MDD DRT method choose not to trigger. This is not exactly a disadvantage, but it has to be noted that the method works best when using raw camera frames instead of compressed images or video.

**Figure 23:**
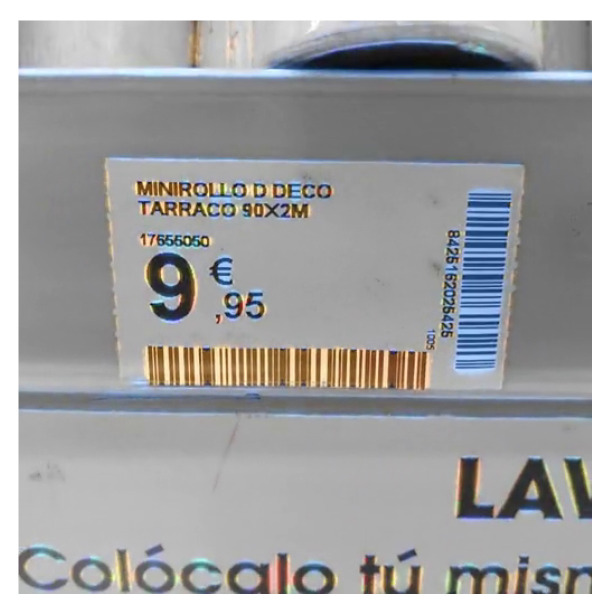
Frame of the video provided in the repository documentation. Available at https://raw.githubusercontent.com/DoMondo/an_encoder_decoder_architecture/master/readme_data/cover.gif, accessed on 25 June 2023.

Another aspect in which our proposed methods distinguish themselves from non-AR-oriented methods is the emphasis on treating the input as a sequence of images, rather than as a still picture. In this sense, the output of many consecutive short-exposure frames is shown in a video provided in the repository, of which an example frame is shown in Figure 23. Again, it is difficult to assess how temporal changes would affect neural network-based methods, but in our method, differential changes in the input induce differential changes in the output, as is desirable, and the marks do not shift or disappear, even in the presence of considerable motion blur and noise due to shooting indoor with short exposure times.

### 6.6. Disadvantages

The main disadvantages of the MDD DRT method are exemplified in the above challenging scenarios and can be summarised as follows:

- Certain alphanumeric characters above or below barcodes, and lines and outlines to the left and right, tend to merge with the barcodes, producing false positives.
- When several adverse circumstances concur, a single instance of a barcode can be split into two connected regions.

It could be argued that neural network-based methods would solve both problems at their root without paying specific attention to them, whereas here, they must be taken into account. However, errors do not arise unpredictably here, while it is often the case when networks are pruned or retrained on different data. With our proposed methodologies, problems can be tackled specifically and with more or less simple methods, depending on the available time margin and severity of the error.

The methods are based on the characterisation of one-dimensional barcodes as sequences of parallel bars, so the detector cannot be used for the detection of two-dimensional codes, such as QR codes.

### 6.7. Conclusions

Considering the computation time cost and the qualitative assessment, the authors chose MDD DRT as the best solution for solving the problem of barcode segmentation for AR. This method allows real-time execution on desktop CPUs. The detection quality is not sacrificed in areas that are particularly relevant in AR-oriented applications, e.g., detection of barcodes affected by motion and lens blur; performance in image sequences acquired in video time; and detection in one-megapixel image streams, with a labelled output of a quarter of the input resolution.

The execution time of the proposed method was measured and compared with state-of-the-art methods. The MDD DRT outperformed other methods, when using a CPU. The accuracy was also measured. The proposed method performed worse than state-of-the-art machine learning methods that use widely available labelled datasets. Regardless, the goodness of the method consisted of the speed and the ability to deal with AR scenarios. For this, a small set of images, representative of these scenarios, was discussed as a qualitative analysis. Finally, synthetic tests were performed to evaluate the robustness of the method.

### 6.8. Future Lines of Research

It is the belief of the authors that a simple morphological closing method with a structuring element with orientation orthogonal to the bars can greatly ameliorate the problems that are now the performance limit of the MDD DRT method, but it is not the object of study of this paper, which is limited to presenting a segmenter that maximises true positives while minimising false negatives. If simple post-processing methods were not enough, it could be considered to improve the voting system of the decoder so that there is no overflow for nearby characters nor discontinuities in the segmentation of codes, but this remains an open line for the future.

On the other hand, the MDD DRT method consists of two parts that could be used separately. This gives rise to the possibility of creating a new detector that is valid for two-dimensional codes and still reuse the decoder. Another future line of work could be to use the decoder in other problems for which an appropriate encoder can be rewritten, such as the depth-from-focus problem [55,56].

The MDD DRT method does not currently meet the time constraints of mobile devices. However, improvements in mobile CPU architectures are so fast that soon the algorithm will be able to run in real time when using the same implementation and a newer CPU. There are two things that can be considered in this regard:

- First, the scheduling of the algorithm in Halide can be improved, since the measurements were manually taken using an auto-scheduler, which gives a schedule that can be improved upon. In addition, the GPU target of SD 888 can also be used.
- Second, more pruning can be made on MDD DRT by modifying two constants: input size and halting stage. For example, starting from 512×512 instead of 1024×1024 and stopping at tile_size = 16 would reduce the execution time to  40% of the original time. How much pruning can be performed without losing too much quality is an assessment that can be performed in follow-up research.

## Figures and Tables

**Table 1:** Output data sizes for the partial DRT algorithm, modified to return data from all the stages and bar_detector. The dimensions of the partial DRT are n_tiles x n_slopes x n_displacements, and the dimensions of the bar_detector are channel x n_tiles x n_tiles, where the channel contains the intensity and the angle planes.

tile_size	$F_{i}$	Partial DRT	Bar Detector
1	$F_{0}$	-	-
2	$F_{1}$	512×3×1024	2×512×512
4	$F_{2}$	256×7×1024	2×256×256
8	$F_{3}$	128×15×1024	2×128×128
16	$F_{4}$	64×31×1024	2×64×64
32	$F_{5}$	32×63×1024	2×32×32

**Table 2:** Output data sizes for the pruned MDD DRT algorithm. The unpool operations include the summation with the original activation functions of the encoder, as it is implemented in the Halide version. The changes resulting from pruning are marked in green.

Block	Operation	Input Size	Output Size
Encoder	mdd_drt_v	1024,1,1024	[1024,3,512] [1024,7,256] [1024,15,128] [1024,31,64] [1024,63,32]
Encoder	mdd_drt_h	1024,1,1024	[1024,3,512] [1024,7,256] [1024,15,128] [1024,31,64] [1024,63,32]
Encoder	mdd_bar_detector_0	[1024,3,512] [1024,3,512]	[6,512,512]
Encoder	mdd_bar_detector_1	[1024,7,256] [1024,7,256]	[14,256,256]
Encoder	mdd_bar_detector_2	[1024,15,128] [1024,15,128]	[30,128,128]
Encoder	mdd_bar_detector_3	[1024,31,64] [1024,31,64]	[62,64,64]
Encoder	mdd_bar_detector_4	[1024,63,32] [1024,63,32]	[126,32,32]
Decoder	unpool_3	[126,32,32] [62,64,64]	[62,64,64]
Decoder	convolutions_3	[62,64,64]	[62,64,64]
Decoder	unpool_2	[62,64,64] [30,128,128]	[30,128,128]
Decoder	convolutions_2	[30,128,128]	[30,128,128]
Decoder	unpool_1	[30,128,128] [14,256,256]	[30,256,256]
Decoder	convolutions_1	[30,256,256]	[30,256,256]
Decoder	unpool_0	[30,256,256] [6,512,512]	[30,512,512]
Decoder	convolutions_0	[30,512,512]	[30,512,512]
Decoder	argmatxth	[30,512,512]	[512,512,3]

**Table 3:** CPU execution time results of our proposed methods compared with a selection of classical and neural network-based methods.

Method	Resolution	Time (ms)
YOLO v5 [28]	1024 × 1024	70
	512 × 512	61
	640 × 480	45
Zamberletti et al. (2013) [16]	640 × 480	130
Creusot and Munawar (2015) [10]	640 × 480	115
Creusot and Munawar (2016) [11]	640 × 480	42
	1080 × 960	116
Zharkov and Zagaynov (2019) [17]	512 × 512	44
MDD DRT	1024 × 1024	21
PS DRT	1024 × 1024	107

**Table 4:** Accuracy metric results for our methods, those in Table 3, and those considered state-of-the-art methods in [24] on two barcode datasets.

Method	Arte-Lab Rotated		WWU Münster	
	$D_{0.5}$	$D_{0.5–0.9}$	$D_{0.5}$	$D_{0.5–0.9}$
EfficientDet [26]	1.000	0.855	0.999	0.782
Faster R-CNN [25]	1.000	0.859	1.000	0.792
Retina Net [27]	1.000	0.876	1.000	0.809
YOLO v5 [28]	0.996	0.935	0.998	0.896
YOLO x [29]	0.970	0.848	1.000	0.813
Zamberletti et al. (2013) [16]	0.805	-	0.829	-
Creusot and Munawar (2015) [10]	0.893	-	0.963	-
Creusot and Munawar (2016) [11]	0.989	-	0.982	-
Hansen et al. (2017) [18]	0.926	-	0.991	-
Zharkov and Zagaynov (2019) [17]	0.989	-	0.980	-
PS DRT	0.886	0.700	0.944	0.732
MDD DRT	0.901	0.783	0.958	0.827

## Data Availability

The images used for evaluating the quality of the results and the code to generate them are available in [51].

## Abbreviations

The following abbreviations are used in this manuscript:

AI	Artificial intelligence
AR	Augmented reality
CNN	Convolutional neural network
CPU	Central processing unit
DRT	Discrete Radon transform
FCN	Fully convolutional network
GPU	Graphics processing unit
SoC	System on a chip
SD	Snapdragon
IoU	Intersection over union
